# Beneath the Top of the Iceberg: Financial Capacity Deficits in Mixed Dementia with and without Depression

**DOI:** 10.3390/healthcare11040505

**Published:** 2023-02-09

**Authors:** Vaitsa Giannouli, Magdalini Tsolaki

**Affiliations:** 1 School of Medicine, Aristotle University of Thessaloniki, 54634 Thessaloniki, Greece; 2 Department of Psychology, University of Western Macedonia, 53100 Florina, Greece

**Keywords:** mixed dementia, depression, financial capacity

## Abstract

Nowadays, controversy exists regarding the influence of comorbid depression on cognition in old age. Additionally, we still know little about the influence of depression in mixed dementia (MD), that is, in cases where there is the co-existence of Alzheimer’s disease and vascular dementia (VaD). Given that the assessment of financial capacity is pivotal for independent living as well as in the prevention of financial exploitation and abuse in old age, in this pilot study, we aimed to examine whether comorbid depression in MD patients can influence financial capacity performance. A total of 115 participants were recruited. They were divided into four groups: MD patients with and without depressive symptoms and healthy elderly without depression as well as older adults suffering from depression. Participants were examined with a number of neuropsychological tests, including the Mini-Mental State Examination (MMSE), Geriatric Depression Scale (GDS-15), and Legal Capacity for Property Law Transactions Assessment Scale (LCPLTAS). The results of this study suggested that financial capacity as measured with LCPLTAS in MD patients was severely impaired when depression co-existed compared to patients suffering only from depression and healthy controls. Deficits in financial capacity in MD and comorbid depression should be a point on which healthcare professionals should focus during neuropsychological assessment in order to prevent financial exploitation.

## 1. Introduction

Mixed/multifactorial dementia (MD) is a condition in which a person has more than one type of dementia, and for the present study, it is defined as the combination of the two most prevalent types of dementia (most frequent in the older population), namely Alzheimer’s Disease (AD) and Vascular Dementia (VaD) and seems to be more common than previously believed in the older adult population [1,2,3]. There is a huge debate regarding the very existence of this heterogeneous disorder, namely the conceptualization and diagnosis of these two entities, and the hypothesized separate or common mechanisms underlying AD and VaD, and so far, the diagnosis of MD is considered to be a diagnostic challenge based on the clinical/neuroimaging criteria of possible AD plus cerebrovascular disease (CVD) [4,5,6].

Due to the lack of consensus on the diagnostic criteria and the heterogeneous neuropathological characteristic of MD, this disorder has not been widely studied [7]. Brain findings highlight the common existence of CVD lesions (e.g., lacunes and white matter lesions) in patients with AD, and typical pathological changes found in AD (e.g., extracellular amyloid plaques and intracellular neurofibrillary tangles) are frequently observed in patients with VaD [7], thus supporting the co-existence of similar brain mechanisms in these two diagnoses.

The fact that an older person may have a combination of two types of dementia means that symptomatology may vary. There is evidence supporting that MD is characterized by lower scores compared to AD patients [8]. More specifically, reduced performance has been found in tests examining basic cognitive domains such as attention [8], memory [9], denomination [9], visuo-construction tasks, and spatial abilities [8], as well as executive functions [10] among patients with MD of mild to moderate severity compared to patients with similar AD severity as those with a diagnosis of AD reported in an additional study [11]. 

One area of focus regarding Instrumental Activities of Daily Living (IADLs), which is of extreme importance for the older population, is financial capacity. Financial capacity is considered to be a broad and complex psychological construct that includes a variety of activities and specific skills (e.g., arithmetic counting coins/currency, paying bills, etc.) and judgment-decision-making skills. Deficits in financial capacity performance have been found in older patients with a diagnosis of VaD [12], AD [13], Parkinson’s Disease with Dementia (PDD) [14], and in amnestic Mild Cognitive Impairment (aMCI) [15]. It is of interest that in the abovementioned groups of patients, the existence of comorbid depressive symptomatology clearly deteriorates further financial performance [12,13,14,15]. 

In addition to that, research has shown deficits in arithmetic and financial problems, which are included in instruments measuring financial capacity [16] per se in patients suffering from fronto-temporal dementia (FTD), and relevant self-overestimations were also reported by the patients regarding their financial performance [17]. This distorted self-awareness (overestimation) is also detected in MCI patients, in mild and moderate AD patients, Parkinson’s disease patients, and in patients with Lewy Body Dementia (LBD) [18,19].

While the research on financial capacity and old age is still emerging, patients with a diagnosis of MD have been largely neglected. So far, only one study has shown that individuals with MD without depressive symptomatology demonstrate similar financial capacity performance with AD patients without depression and similar Mini-Mental State Examination (MMSE) scores [16].

Biologically, brain areas such as the medial frontal and anterior temporal cortices’ decreased activity are related to impaired self-awareness in AD patients [20,21], and inaccurate self-evaluations of cognitive domains, such as memory which is involved in financial capacity performance are controlled by the prefrontal cortex [22]. Of relevant interest is also the atrophy in the frontal and parietal lobes [23] and, more specifically, the angular gyrus in the left parietal lobe in MCI (MCI) [24,25] as well as in mild AD patients [21].

Given all the above brain areas, which are not only affected in AD and MCI, but in many cases of MD patients, the aim of this research is to examine for the first time financial capacity performance in MD patients, and more specifically in MD patients with or without comorbid depressive symptomatology compared to healthy older adults with and without depression.

## 2. Materials and Methods

In the current study, 115 Greek older adults (n = 74 women; n = 41 men) were recruited from the Memory Clinic of Papanikolaou General Hospital and the Greek Alzheimer Association of Alzheimer’s Disease and Related Disorders (GAADRD), Thessaloniki, Greece. The diagnoses of the patients were supported by a consensus of specialized neurologists, geriatric psychiatrists, and neuropsychologists after neurological examination and neuropsychological assessment, medical history, neuroimaging (CT and/or MRI), and blood tests. Thirty participants had a diagnosis of MD (n = 16 women; n = 14 men) characterized as moderate cognitive impairment (based on Mini-Mental State Examination (MMSE) scores), with no depressive symptomatology present based on a useful self-report tool used worldwide as well as in the Greek older population with a cutoff score of 6–7 points [26]; the 15-item Geriatric Depression Scale (GDS-15) score for all included participants was <6 [26] (M_GDS-15_ = 1.70; SD = 1.51). A total of 25 participants had a diagnosis of moderate MD with depressive symptomatology (n = 13 women; n = 12 men) according to their self-reported depressive symptomatology (M_GDS-15_ = 9.84, SD = 2.77). 

A total of 30 community-dwelling older adults (n = 23 women; n = 7 men) (M_GDS-15_ = 0.56, SD = 1.27), with no diagnosis related to cognitive deficits and with GDS-15 scores < 6, were approached following purposeful sampling (regarding their gender, age, and education characteristics) and were also tested as a control group. In addition to that, 30 older patients suffering only from depression (n = 22 women; n = 8 men) were also tested (M_GDS-15_ = 10.10, SD = 1.84).

Regarding the two groups characterized as MD depressed and solely depressed, at least two of the following criteria had to be met, following similar previous studies [12,13,14,15]): (1) self-reported active depression in the last two years, (2) depression/dysphoria symptoms as reported on the Neuropsychiatric Inventory Questionnaire (NPI-Q), (3) clinically depressed mood based on clinician interview, (4) a GDS-15 score of at least six, and (5) depressive symptomatology reported by the participants’ family or other people (caregivers, healthcare experts, friends) for a maximum of 12 months period. 

Healthy controls were matched with the patient groups (MD, MD depressed, and only depressed individuals) regarding age [F(3, 110) = 1.363, *p* = 0.258], years of education [F(3, 110) = 0.356, *p* = 0.785], and gender [χ^2^(3) = 6.685, *p* = 0.083] (see Table 1). 

Participants in all four groups were excluded as in similar studies [12,13,14,15] if the following criteria were present: a history of substance abuse, previous traumatic brain injury (TBI) and related neurosurgical interventions, concomitant serious medical illness (significant visual and/or auditory impairment not corrected sufficiently by visual/auditory aids), a history of other neurologic or psychiatric disorder that may interfere with the patient’s neuropsychological performance. For the MD group, 1 individual was excluded; for the MD group with depressive symptomatology, 2 individuals were excluded; for the group with a diagnosis of depression, 1 individual was excluded; for the healthy group, 1 individual was excluded (their scores are not presented and not included in the final analyses of this paper).

An extensive neuropsychological test battery was administered in order to assist in diagnosis by assessing basic cognitive abilities such as attention, working memory, abstraction, inhibition, fluency, verbal learning and memory, visual memory, visuospatial skills, and psychomotor speed. These tests were the Mini-Mental State Examination (MMSE), Test of Everyday Attention (ΤΕA), Trail Making Test (TMT)-Parts A and B, Rey-Osterrieth Complex Figure Test (ROCF)-copy condition and immediate and delayed recall conditions of the complex design, Rey Auditory Verbal Learning Test (RAVLT), Rivermead Behavioural Memory Test (RBMT), Verbal Fluency Task, Neuropsychiatric Inventory (NPI), Instrumental Activities of Daily Living (ΙADL), Clinical Dementia Rating (CDR), and Functional-Cognitive Assessment Scale (FUCAS). Financial capacity was assessed with the Legal Capacity for Property Law Transactions Assessment Scale (LCPLTAS), which consists of seven main domains: basic monetary skills, cash transactions, bank statement management, bill payment, financial conceptual knowledge, financial decision-making, and knowledge of personal assets [16]. LCPLTAS is based on the Financial Capacity Instrument introduced by Marson and follows the same conceptual framework. More specifically, older adults are asked to complete a number of tasks ranging from simple tasks (such as naming coins, performing basic mathematical operations, defining verbally financial concepts, and making statements about their personal assets) to more complex ones (such as deciding on hypothetical financial scenarios and explaining their thinking steps and relevant decision-making). All participants were examined during the same semester (during the period from October 2013 to March 2014). There was only one version of LCPLTAS administered at one time point by the same neuropsychologist. Neuropsychological testing took place individually for each participant at the same location (a quiet soundproof room without distracting stimuli), and for all participants, evaluations were conducted during morning hours.

All participants provided written informed consent prior to study participation and were not compensated for their participation in the study. They agreed to participate voluntarily and that they could withdraw their participation at any time without providing any reason and without any cost. The study protocol was approved by the Ethics Committee of the Aristotle University of Thessaloniki (as part of a larger study [16]) and followed the declaration of Helsinki. 

### Statistical Analysis

Statistical analysis was performed with the SPSS (IBM Corp (2016) IBM SPSS Statistics for Windows (Version 24)). Descriptive statistics and one-way analyses of variance (ANOVAs) were conducted to examine possible differences in demographic factors and cognitive test scores of the four groups (MD patients, patients with MD and comorbid depression, healthy controls, and patients diagnosed only with depression). In addition, Pearson’s correlations between MMSE (as this has been found to be one of the best correlates of LCPTLAS in prior research [13,14,15,16,17,18]) and LCPTLAS were performed. Post-hoc Tamhane’s T2 are presented in order to explore significant differences, and the effect size of the differences was measured with eta squared. Probability values < 0.05 (two-tailed) were considered statistically significant.

## 3. Results

A strong positive correlation was found between the MMSE score and the LCPTLAS for the whole sample (r = 0.958, *p* < 0.001). This indicates that high MMSE scores (which reflect high cognitive ability) correlate in a statistically significant way with high scores on LCPTLAS, which depicts high financial ability. For the whole sample, no other statistically significant correlation was found between LCPTLAS and education (in years) (r = 0.089, *p* = 0.348) as well as for LCPTLAS and age (in years) (r = 0.061, *p* = 0.520).

One-way ANOVA revealed (as expected) significant differences and large effect sizes for the MMSE scores (F(3, 108) = 140.398, *p* < 0.001, η^2^ = 0.768). Comparisons following one-way ANOVA for financial capacity as examined by LCPTLAS showed statistically significant differences with the healthy controls without depression performing best, followed by patients suffering only from depression, moderate MD patients without depression, and MD patients with depression (F(3, 110) = 181.391, *p* < 0.001, η^2^ = 0.831) (see Table 1).

**Table 1:** Demographics, MMSE, GDS-15, and LCPTLAS scores for the four groups.

Variables	Diagnostic Group	Mean	SD	Minimum	Maximum
Age	MD	78.62	7.18	61.00	89.00
	MD depression	75.84	6.25	66.00	84.00
	Healthy	76.16	6.00	65.00	92.00
	Depressed	74.96	8.96	47.00	98.00
Education years	MD	7.96	5.03	1.00	24.00
	MD depression	8.96	4.60	3.00	16.00
	Healthy	8.30	4.48	1.00	17.00
	Depressed	9.03	4.40	2.00	16.00
MMSE	MD	17.00	5.58	7.00	26.00
	MD depression	15.84	3.78	11.00	24.00
	Healthy	29.13	0.43	28.00	30.00
	Depressed	28.36	1.42	26.00	30.00
GDS-15	MD	1.70	1.51	0.00	5.00
	MD Depression	9.84	2.77	6.00	15.00
	Healthy	0.56	1.27	0.00	4.00
	Depressed	10.10	1.84	7.00	13.00
LCPTLAS	MD	54.17	31.74	20.00	133.00
	MD Depression	45.64	19.05	26,.00	93.00
	Healthy	143.56	0.85	141.00	144.00
	Depressed	131.13	15.89	103.00	144.00

## 4. Discussion

The above findings provide support for the first time for a significant impairment in financial capacity in cases of dual mixed pathology, such as MD (AD + vascular) with comorbid depression. The current study investigated performance in MD patients with and without comorbid depressive symptomatology and supports that both diagnostic groups are characterized by financial incapacity and that both groups clearly differentiate from non-depressed matched participants. Age (in years) and educational level (in years) of participants were not found to correlate with financial performance in LCPTLAS in this sample. This is in contrast to previous findings in healthy aging that support a negative influence of advancing age on financial capacity [27], as well as findings regarding education and the fact that a lower educational level negatively affects financial capacity performance in MCI patients [28]. Nevertheless, the current results confirm previous findings regarding the negative influence of depression on financial capacity performance in other groups of older adults suffering from neurocognitive disorders, such as PDD, VD, and MCI [12,13,14,15]. It is of interest that this detrimental influence of depressive symptomatology is corroborated not only by linear analyses but also by advanced statistical methods based on catastrophe theory (nonlinear models) for the elder population [29]. This negative influence has also been supported in longitudinal designs measuring depression levels at different time points [30], a finding that is for the first time supported in this cross-sectional study of MD patients. In addition to that, the finding of a strong positive correlation between MMSE and LCPTLAS highlights the importance of MMSE scores as relevant to financial capacity assessments [12,13,14,15,16,31,32]. 

This is an interesting point as both the MMSE as well as the specifically designed tool for financial capacity assessment (LCPTLAS) could both be used as neuropsychological indicators that are easy to administer and act as important sources of information for everyday functioning as well as sources to build personalized interventions against financial abuse in older adult populations [33,34]. 

Although the current study has several strengths, such as the exclusion of demographic influences that could play the role of confounders, this study also presents some limitations, which should be taken into consideration in future research endeavors, such as the small sample size, the slightly unequal size of the subgroups, and the fact that demented patients were not examined with post mortem autopsy (so the exact brain pathology could be documented in detail). Additionally, variables such as previous stressful life events or prior high levels of depression [35], the behavioral and psychological symptoms of dementia (BPSD) [36], genetic information about the participants (e.g., APOE e4) [37], as well as the simultaneous administration and use of scores coming from other financial capacity instruments [38] were not entered into the analyses. 

## 5. Conclusions

This is the first ever reported study of its kind to show that depression has a clearly differentiating power on financial capacity performance in the group of moderate MD patients. Although none of the participants was characterized as legally incapacitated, thus, officially, there was no legal decision depriving them of their right to make financial decisions, for both groups of MD and MD depressed patients, their family members responded that they unofficially made all relevant financial decisions on behalf of the older adults, something that corroborates the general tendency in Greece to consider as stigma the durable power of attorney for financial decisions, the valid wills as well as the living wills of older adults [39]. Of course, having caregivers make financial decisions for MD patients seems to be a solution, but this is done unofficially as the caregivers were not appointed by the court and, thus, they are not legal guardians, something that still renders older people vulnerable to financial exploitation. Given that financial capacity deficits in MD patients should not be underestimated, future research should focus on unraveling the neurobiological substrate of financial incapacity when depression is present in MD. Furthermore, healthcare professionals should be aware of the financial effects of dementia and depression, and although they are not trained in financial management, they can still inform caregivers so they can make changes by taking over the financial affairs of older adult patients only after being officially determined by a court as legally incapacitated for financial decision-making.

## Data Availability

The data presented in this study are available on request from the corresponding author. The data are not publicly available due to privacy issues.
